# Physical therapy vs. internet-based exercise training (PATH-IN) for patients with knee osteoarthritis: study protocol of a randomized controlled trial

**DOI:** 10.1186/s12891-015-0725-9

**Published:** 2015-09-28

**Authors:** Quinn I. Williams, Alexander H. Gunn, John E. Beaulieu, Bernadette C. Benas, Bruce Buley, Leigh F. Callahan, John Cantrell, Andrew P. Genova, Yvonne M. Golightly, Adam P. Goode, Christopher I. Gridley, Michael T. Gross, Bryan C. Heiderscheit, Carla H. Hill, Kim M. Huffman, Aaron Kline, Todd A. Schwartz, Kelli D. Allen

**Affiliations:** Thurston Arthritis Research Center, University of North Carolina at Chapel Hill, 3300 Thurston Bldg., CB# 7280, Chapel Hill, NC 27599 USA; Department of Medicine, University of North Carolina at Chapel Hill, 125 MacNider Hall CB# 7005, Chapel Hill, NC 27599 USA; Visual Health Information, Inc., Tacoma, WA USA; Injury Prevention Research Center, University of North Carolina at Chapel Hill, Chapel Hill, NC USA; Comprehensive Physical Therapy Center, Chapel Hill, NC USA; Department of Epidemiology, University of North Carolina at Chapel Hill, Chapel Hill, NC USA; Department of Orthopedic Surgery, Division of Physical Therapy, Duke University Medical Center, Durham, NC USA; Advanced Physical Therapy of Smithfield, Smithfield, NC USA; Division of Physical Therapy, Department of Allied Health Sciences, University of North Carolina at Chapel Hill, Chapel Hill, NC USA; Department of Orthopedics and Rehabilitation, University of Wisconsin-Madison, Madison, WI USA; Department of Medicine, Division of Rheumatology, Duke University Medical Center, Durham, NC USA; Physical Medicine and Rehabilitation Service, Durham VA Medical Center, Durham, NC USA; Department of Biostatistics, Gillings School of Global Public Health, University of North Carolina at Chapel Hill, Chapel Hill, NC USA; School of Nursing, University of North Carolina at Chapel Hill, Chapel Hill, NC USA; Center for Health Services Research in Primary Care, Durham VA Medical Center, Durham, NC USA

**Keywords:** Osteoarthritis, Knee, Physical Therapy, Internet, Physical Activity

## Abstract

**Background:**

Physical activity improves pain and function among individuals with knee osteoarthritis (OA), but most people with this condition are inactive. Physical therapists play a key role in helping people with knee OA to increase appropriate physical activity. However, health care access issues, financial constraints, and other factors impede some patients from receiving physical therapy (PT) for knee OA. A need exists to develop and evaluate other methods to provide physical activity instruction and support to people with knee OA. This study is examining the effectiveness of an internet-based exercise training (IBET) program designed for knee OA, designed by physical therapists and other clinicians.

**Methods/Design:**

This is a randomized controlled trial of 350 participants with symptomatic knee OA, allocated to three groups: IBET, standard PT, and a wait list (WL) control group (in a 2:2:1 ratio, respectively). The study was funded by the Patient Centered Outcomes Research Institute, which conducted a peer review of the proposal. The IBET program provides patients with a tailored exercise program (based on functional level, symptoms, and current activity), video demonstrations of exercises, and guidance for appropriate exercise progression. The PT group receives up to 8 individual visits with a physical therapist, mirroring standard practice for knee OA and with an emphasis on a home exercise program. Outcomes are assessed at baseline, 4 months (primary time point) and 12 months (to assess maintenance of treatment effects). The primary outcome is the Western Ontario and McMaster Universities Osteoarthritis Index, and secondary outcomes include objective physical function, satisfaction with physical function, physical activity, depressive symptoms and global assessment of change. Linear mixed models will be used to compare both the IBET and standard PT groups to the WL control group, examine whether IBET is non-inferior to PT (a treatment that has an established evidence base for knee OA), and explore whether participant characteristics are associated with differential effects of IBET and/or standard PT. This research is in compliance with the Helsinki Declaration and was approved by the Institutional Review Board of the University of North Carolina at Chapel Hill.

**Discussion:**

The IBET program could be disseminated widely at relatively low cost and could be an important resource for helping patients with knee OA to adopt and maintain appropriate physical activity. This trial will provide an important evaluation of the effectiveness of this IBET program for knee OA.

**Trial registration:**

NCT02312713

## Background

Osteoarthritis (OA) is one of the most common chronic health conditions and a leading cause of pain and disability among adults [1–3]. Knee OA is particularly common, with recent data indicating that 45 % of people may develop symptomatic knee OA in their lifetime [4]. Because of the forecasted growth in the U.S. older adult population, the prevalence of knee OA is expected to rise dramatically over the next several decades [5]. In addition, research indicates that knee OA is occurring earlier in life, affecting younger adults more often than in previous years, likely due to increased rates of obesity and joint injury [6, 7]. The rising prevalence and earlier occurrence of knee OA highlight the need for effective disease management strategies.

Many studies have confirmed that physical activity improves pain, function, and other key outcomes among patients with knee OA [8, 9]. Based on this evidence, exercise is considered a cornerstone of managing knee OA [10–13]. However, the majority of adults with OA are physically inactive [14, 15], and efforts are needed to promote physical activity in these patients [16–18]. Physical therapists can play a key role in helping patients with OA improve their physical activity and related outcomes. Physical therapy (PT) is also consistently recommended as component of knee OA treatment in professional guidelines [10–13]. However, health care access-related issues can impede some patients with OA from receiving this important component of care. In medically underserved areas, PT services can be limited or lacking entirely. Some patients with OA lack insurance coverage, and for many others copayments make receipt of PT cost-prohibitive. These issues are particularly salient for individuals with low socioeconomic status, who also bear a greater burden of OA [19–21]. These issues highlight a need for additional methods to provide instruction in and support for physical activity and self-management for patients with knee OA.

The internet has been increasingly used to deliver physical activity and other behavioral programs [22–24]. While face-to-face PT visits and other physical activity programs clearly have value, several important opportunities are associated with internet-based delivery, including the capacity for widespread dissemination at relatively low cost and convenience for users. To date little research exists on internet-based physical activity programs for individuals with OA [25, 26], and few studies of this kind have focused on older adults who comprise a large proportion of this patient group [27, 28]. This manuscript describes the protocol of a randomized clinical trial examining the effectiveness of an internet-based exercise training program (IBET) and standard PT for patients with knee OA, both compared to a wait list control group. The study will also examine whether the novel IBET program is as effective as PT, which already has an established evidence base for knee OA. Another aim of this study is to examine whether patient characteristics are associated with differential improvement IBET and/or standard PT groups. This information can help guide patients toward the treatment option that may be most beneficial for them. The specific aims and hypotheses of this study are:

### Aim #1

Compare the effects of IBET and standard PT for knee OA on short-term (four-month) patient-centered outcomes, vs. a wait list (WL) control group.

#### Hypothesis 1 (H1; Superiority)

Patients who receive either IBET or standard PT will have clinically relevant improvements in pain, stiffness, and function, measured by the Western Ontario and McMasters Universities Osteoarthritis Index (WOMAC) at four-month follow up, compared with patients in the WL control group.

#### Hypothesis 2 (H2; Non-Inferiority)

IBET will be non-inferior (e.g., achieve comparable outcomes) to standard PT at four months, indicated by a mean WOMAC score less than five points higher (worse) than standard PT.

### Aim #2

Compare the effects of IBET and standard PT for knee OA on longer-term (twelve-month) patient-centered outcomes, vs. a WL control group.

#### Hypothesis 3 (H3; Superiority)

Patients who receive either IBET or standard PT will have clinically relevant improvements in WOMAC scores at twelve -month follow-up, compared with the WL control group.

#### Hypothesis 4 (H4; Non-Inferiority)

IBET will be non-inferior to standard PT at twelve months, indicated by a mean WOMAC score less than five points higher (worse) than standard PT.

### Aim #3

Examine whether individual patient characteristics (particularly age and baseline functional status) are associated with differential improvement in IBET and/or standard PT.

## Methods

This study was reviewed and approved by the Institutional Review Boards of the University of North Carolina at Chapel Hill and Duke University Medical Center.

### Study design and setting

**Fig. 1:**
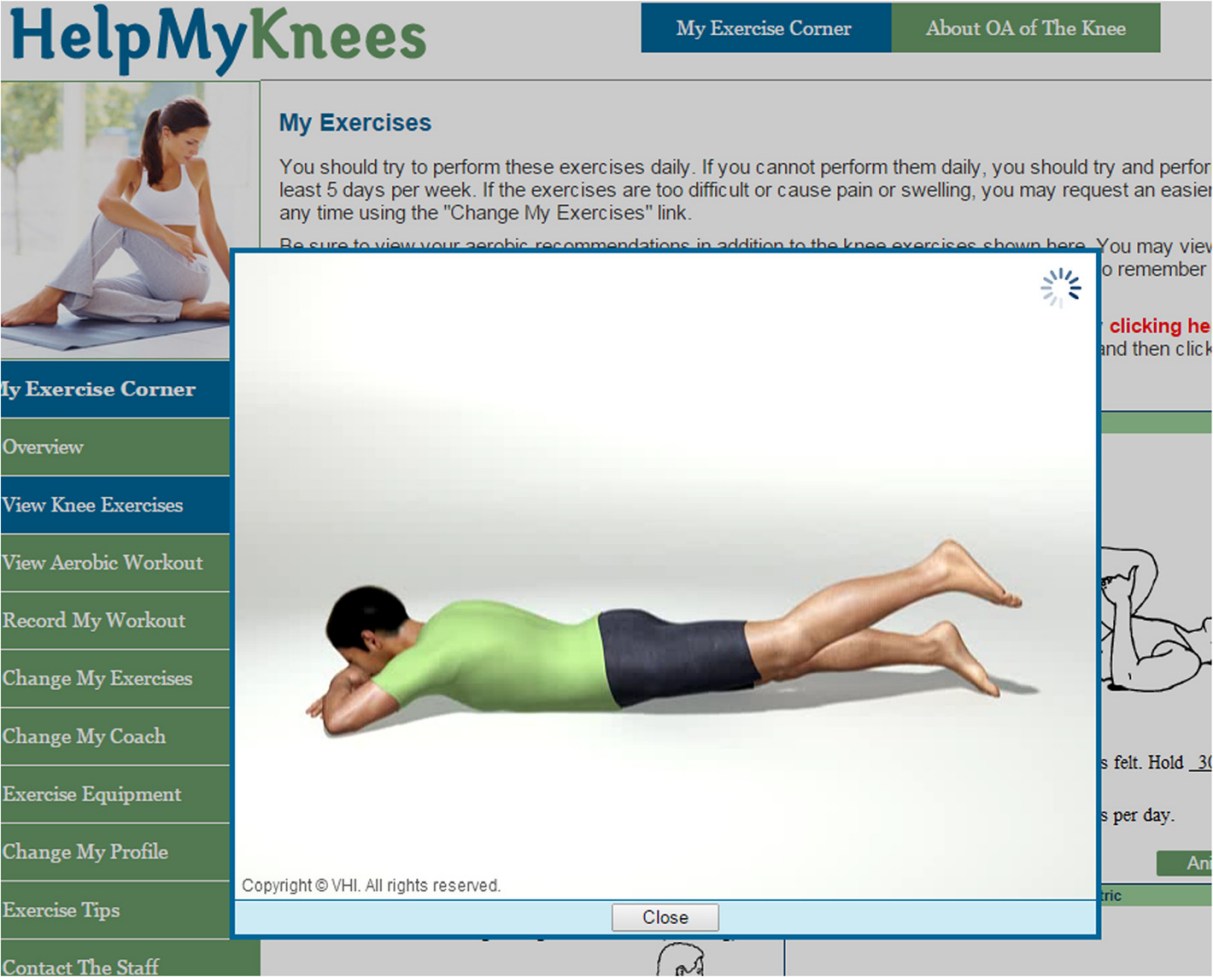
Demonstration of Exercises from Internet-Based Program

The PhysicAl THerapy vs INternet-Based Exercise Training for Patients with Knee Osteoarthritis (PATH-IN) study is a randomized controlled trial with participants assigned to three groups: Standard PT for knee OA, IBET for knee OA, and wait list (WL) control, with allocation of 2:2:1, respectively (Fig. 1). Participants are stratified by enrollment source (described below) to ensure groups are balanced in this respect. The three measurement time points are at baseline, 4 month follow-up, and 12 month follow-up. A 4 month duration for the initial intervention period was chosen because this is an adequate time period to observe meaningful changes in pain and function in the context of OA [29]. The 12 month assessment will evaluate whether there are sustained effects beyond the initial intervention period. The IBET group will continue to have access to the website between the 4 month and 12 month assessment points. Following completion of the 12 month assessments, participants assigned to the WL control group will receive two PT visits plus access to the IBET program. No study data will collected from WL participants after they have received these interventions. Rather, provision of these interventions is for ethical considerations, providing these participants with access to the study OA therapies. Participants in all study groups will continue with their usual medical care for OA during the full study period.

Study participants are being enrolled though active recruitment methods and advertisements for self-referral from two geographic regions and settings, to enhance generalizability. First, patients are being identified from the University of North Carolina at Chapel Hill (UNC) and surrounding area. UNC has a large non-profit healthcare system that provides both primary and specialty care. Over 800,000 people receive outpatient care at UNC clinics annually. Second, individuals with symptomatic knee OA are being identified from the Johnston County Osteoarthritis Project (JoCo OA), an ongoing study in rural North Carolina [30]. African Americans and individuals 60 years of age and older comprise about 20 % and 17 % of the county’s population, respectively. Households with limited education and lower income are common in Johnston County, with 35 % of individuals over age 25 having less than a high school diploma and 30 % of jobs involving manufacturing, service or farming.

### Participant eligibility criteria

Participants must meet the following criteria for at least one knee:

- Diagnosis of Knee OA. For participants enrolled from the UNC healthcare system database, knee OA is identified from electronic medical records. For participants enrolled from the JoCo OA study, knee OA is identified from previous study-based radiographs. For self-referred patients, this is identified based on self-report, including items based on the American College of Rheumatology criteria for knee OA [31].
- Current Joint Symptoms. Participants must indicate having pain, aching or stiffness in one or both knees on most days of the week

Exclusion criteria are shown in Table 1.

**Table 1:** Exclusion criteria

No regular internet access
Currently meeting Department of Health and Human Services Guidelines for Physical Activity [32]
Currently completing series of physical therapy visits for knee OA
Diagnosis of gout in the knee, rheumatoid arthritis, fibromyalgia, or other systemic rheumatic disease
Severe dementia or other memory loss condition
Active diagnosis of psychosis or current uncontrolled substance abuse disorder
On waiting list for arthroplasty
Hospitalization for a stroke, heart attack, heart failure, or had surgery for blocked arteries in the past 3 months
Total joint replacement knee surgery, other knee surgery, meniscus tear, or ACL tear in the past 6 months
Severely impaired hearing or speech
Unable to speak English
Serious or terminal illness as indicated by referral to hospice or palliative care
Other health problem that would prohibit participation in the study
Nursing home residence
Current participation in another OA intervention study
Fall history deemed by a study physical therapist co-investigator to impose risk for potential injury with participation in a home-based exercise program

### Recruitment, enrollment and randomization

Two general methods of recruitment are being used. First, flyers and brochures and other forms of advertisement are being posted within UNC and the surrounding community, as well as in Johnston County. UNC health care providers may also give brochures to their patients. Second, patients are actively being recruited based on UNC medical records and JoCo OA data. Individuals who meet initial eligibility criteria from these two sources are mailed introductory letters. All potential participants, regardless of recruitment source, are screened for additional eligibility criteria via telephone. Individuals who meet eligibility criteria and are interested in participating are asked to meet a study team member to complete consent, HIPAA authorization, and baseline assessments. Following baseline assessments, participants are given their randomization assignment by the project coordinator via telephone. Randomization is based on a computer generated sequence maintained by the project statistician and programmer. Following randomization, participants assigned to the standard PT group are scheduled for their initial visit, and participants assigned to the IBET group are given instructions and an individual code to access the website, along with ankle weights and resistance bands to facilitate the exercises. Participants randomized to the WL group are informed they will be provided with two PT visits and access to the IBET website after their follow-up assessments are complete.

### Study interventions

#### Internet-based exercise training program

The IBET program used in this study was developed by Visual Health Information and a multidisciplinary team, including physical therapists, physicians and patients; details of the program have been described previously [26]. Visual Health Information did not provide any financial support for this study. A pilot study of this program showed that after eight weeks of use, participants with knee OA experienced significant and clinically meaningful improvements in pain, stiffness and function. The following describes general features of the IBET program:

##### Tailoring of exercises

When participants first log into the IBET site, they complete a brief set of measures regarding their pain, function, and current activity. This includes the modified Short Form of the Western Ontario and McMaster Universities Osteoarthritis Index (mSF-WOMAC). Based on these data, an algorithm is used to assign participants to one of 7 different exercise levels that range in difficulty. Each exercise level includes stretching and strengthening exercises that were judged appropriate for a given functional level by a multidisciplinary group of clinicians [26]. Exercise routines are randomly generated from the participant’s assigned level and always include both stretching and strengthening exercises that target multiple muscle groups, with a focus on the lower extremity and knee function. The initial assigned exercise routine also includes recommendations for amount and intensity of aerobic exercise.

In addition to tailoring the initial exercise routine, the IBET program collects participant information on an ongoing basis to facilitate appropriate progression. At the end of each session participants are given the option to move to a harder or easier exercise level at the next session.

When patients opt for exercise progression, they are prompted to complete the mSF-WOMAC. If their score is worse than the previous administration of the scale, they are not yet advanced to the next exercise level but are instead given a new exercise routine at their current level. If their mSF-WOMAC score is equal to or better than the previous administration, they are allowed to progress along the exercise continuum. As patients progress to more difficult levels, their exercise routine may contain more difficult exercises, previously assigned exercises with increased weight, more sets or repetitions, or an increase in the number of exercises. If patients request an easier exercise routine at any point, they are first advised that slight increases in pain or discomfort are common when beginning an exercise program, and they may want to try icing and rest and then resume their current exercise program but consider reducing the frequency initially. However, participants may still choose to receive a new exercise program immediately at an easier level. In addition, if patients have difficulty with any particular exercises (e.g., it is painful or they cannot get into the required position), the system provides an option to exchange that exercise for another within the same level. Participants may also opt to change their entire exercise routine within the same level.

##### Video display of exercises

In addition to static photos and instructions for each exercise, the IBET includes videos that demonstrate correct performance (Fig. 1). This is important for maximizing benefits and reducing injury risk. Participants can select the gender and race of the animated model that shows correct exercise performance.

##### Pain monitoring

After each session, participants are asked to report whether their pain increased following their exercises. If participants report increased pain for three consecutive sessions without requesting a lower exercise level, they are sent an email suggesting they consider trying an easier exercise level. If patients do not record having increased pain for two weeks and have not requested an increase in exercise level in that interval, they are sent an email suggesting they consider trying a more difficult level.

##### Automated reminders

If participants do not interact with the IBET website for seven days, they receive an email encouraging them to access the website, and, most importantly, to remain physically active.

##### Tracking progress

**Fig. 2:**
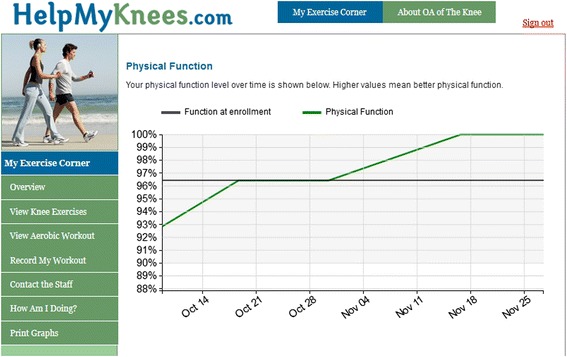
Tracking of Self-Reported Physical function from Internet-Based Program

The IBET website provides participants with graphs of their pain, physical function, and exercise over time, so they can visualize their progress (Fig. 2).

##### Other features

The IBET website also includes general tips for exercise with knee OA, as well as other suggestions for self-management strategies.

Participants in this study are asked to access the IBET site as soon as they are randomized and to continue through the 12-month follow-up assessment. In accordance with current Department of Health and Human Services and other guidelines for physical activity among older adults [32], participants are encouraged to complete strengthening and stretching exercises, guided by the IBET website, at least 3 times per week. Also in accordance with physical activity guidelines, participants are informed that it is safe and appropriate to perform aerobic exercises daily, or as often as possible, guided by the recommendations on the IBET website for their current exercise level. An overall guiding principle is that participants are encouraged to be as physically active as their abilities, health conditions, and pain allow.

Participants are given a telephone number to contact a study team member if they need technical support regarding the website or have questions about their exercise program. The IBET website also includes a link to contact the study team via email if they have questions. The nature and frequency of these contacts are being documented to contribute to the overall evaluation of this internet-based resource.

#### Physical therapy intervention

The standard PT intervention is modeled after typical elements of care provided to patients with knee OA [33], including:

1. evaluation of specific areas of weakness or inflexibility, evaluation of mobility, balance, function, knee alignment, and possible limb length inequality;
2. evaluation of the need for mobility aids, knee braces, patellar taping (for edema, pain management, joint alignment and/or proprioception), heel lifts, shoe wedges and other footwear modifications;
3. instruction in an appropriate home exercise program (including strengthening, stretching/range of motion, and aerobic exercises);
4. instruction in strategies for pacing daily activities and protecting joints, including proper movement patterns to decrease stresses and strains;
5. manual therapy, if appropriate (e.g. joint mobilization, stretching, soft tissue mobilization);
6. modalities for pain management (if appropriate).

Emphasis is placed on the home exercise program, which is initiated at the first visit and incorporated into each subsequent visit. To mirror standard clinical practice, physical therapists are permitted to tailor visits to patients’ needs and functional limitations. Also to mirror clinical practice, some variability is permitted with respect to number of PT visits. Based on a typical range of outpatient PT visits for knee OA, study participants can receive up to 8 one-hour sessions. At the first visit, physical therapists complete a standardized, electronic evaluation form, along with documentation of any treatment provided during that visit. At subsequent visits, physical therapists complete an electronic progress note form that includes documentation of treatment components provided (including therapeutic exercise, balance/neuromuscular education, manual therapy, gait or stair training, shoes/wedges, and modalities).

Physical therapists in multiple clinics proximal to UNC and in Johnston County, who have experience in treating patients with knee OA, are delivering this arm of the study. All study physical therapists are trained by co-investigators in the standard PT arm elements, as well as use of the study database. Physical therapist co-investigators (Golightly, Goode) are observing a portion of visits (as well as viewing electronic evaluation forms and progress notes) for each study therapist to ensure fidelity and adherence to the study protocol. Appendix shows the general guidance given to study physical therapists for the delivering the intervention, and this form is also used as a checklist by physical therapist co-investigators when monitoring fidelity.

### Measures

All study assessments are conducted by trained research assistants blinded to participants’ randomization assignment. Assessments are typically conducted in person, but there is some allowance for telephone-based follow-up assessments in cases where participants are unable to return to the study site. Participants are paid $30 for completion of assessments at each time point.

#### Primary outcome: Western Ontario and McMasters Universities Osteoarthritis Index (WOMAC)

The WOMAC is a measure of lower extremity pain (5 items), stiffness (2 items), and function (17 items) [34, 35]. All items are rated on a Likert scale of 0 (no symptoms) to 4 (extreme symptoms). The reliability and validity of the WOMAC total score and subscales have been confirmed [35], and this scale has been widely used in trials of behavioral interventions for patients with knee OA, confirming its sensitivity to change for these interventions.

#### Secondary outcomes

##### Satisfaction with physical function scale

This is a validated 5-item questionnaire that assesses patients’ satisfaction with their ability to complete basic functional tasks that are often affected by lower extremity OA, including stair-climbing, walking, doing housework (light and heavy, and lifting and carrying [36]. All items are rated on a 7 point scale ranging from Very Dissatisfied (-3) to Very Satisfied (+3).

##### Objective physical function

These tests assess aspects of daily function that require lower extremity strength, and that are often impacted by knee or hip OA. The Four-Stage Balance Test includes side by side stand, semi-tandem stand, tandem stand, and unilateral stand [37, 38]. Each balance tests is measured for up to ten seconds. Other tests include the 30-second chair stand [39], the Timed Up and Go Test [40, 41], and a two-minute step test [42].

##### Depressive symptoms – Patient Health Questionnaire-8 (PHQ-8)

The PHQ-8 is an 8-item survey derived from the Primary Care Evaluation of Mental Disorders (PRIME-MD) diagnostic tool, and consists of items corresponding to the depression criteria listed in the *Diagnostic and Statistics Manual Fourth Edition* (DSM-IV) [43]. Each of the 8 questions is scored as 0 (not at all) to 3 (nearly every day), so that total scores range from 0 to 24.

##### Patient global assessment of change

This scale evaluates participants’ perspectives on overall changes in their joint pain during the study period. This single-item measure asks participants to describe their change in pain on a 7-point rating scale with the following options: “very much improved,” “much improved,” “minimally improved,” “no change,” “minimally worse,” “much worse,” and “very much worse.”

##### Physical activity scale for the elderly

The Physical Activity Scale for the Elderly (PASE) is a self-report, 12-item scale that measures occupational, household, and leisure activities during a 1-week period [44]. This scale was particularly developed for use among older adults and is therefore appropriate in a study of patients with knee OA, who typically have more limited physical activity than the general population.

##### Additional self-report physical activity items

To further assess purposeful exercise behaviors, participants are asked to report the number of times and minutes per week, on average, they are completing strengthening, stretching, and aerobic exercises

##### Use of IBET website/Number of PT visits attended

The frequency of participants’ use of the IBET website will be logged within the site. For patients in the Standard PT group, the number of visits attended is documented.

#### Process measures

Two key process measures are being assessed, which have been associated with change in physical activity behavior. These provide understanding of potential mechanisms underlying any observed improvement in the IBET and/or standard PT interventions.

##### Self-efficacy for exercise scale

The Self-Efficacy for Exercise Scale assesses individuals’ confidence in engaging in exercise in 9 different situations that could present barriers (including having pain when exercising) [45]. For each situation, individuals are asked to rate their confidence in being able to exercise 3 times a week for 20 min each time, on a scale of 0 (not confident) to 10 (very confident). Validity of this measure was confirmed by expected associations with actual exercise, as well as physical and mental health.

##### Social support for exercise scale

Sallis et al. [46] this scale includes 13 items that assess the frequency with which friends and family members (separately) engage in behaviors that may either support exercise (e.g., “Gave me encouragement to stick with my exercise program”) or discourage exercise (e.g., “Complained about the time I spend exercising”). All items are measured on a scale of 1 (none) to 5 (very often). The scale has shown acceptable test-retest reliability and internal consistency reliability. In addition, the scale was correlated with exercise habits, providing evidence of concurrent criterion-related validity [46].

#### Demographic and clinical characteristics

Participant characteristics include age, race/ethnicity, gender, household financial state (with low income defined as self-report of “just meeting basic expenses” or “don’t even have enough to meet basic expenses”), education level, work status, marital status, internet use/comfort, health literacy, body mass index (BMI), joint involvement (i.e., report of all joints affected by arthritis), duration of OA symptoms, general self-rated health, and comorbid illnesses (Self-Administered Comorbidity Questionnaire [47]).

#### OA treatment use

Participants’ OA treatment use is being assessed at each time point, via self-report, to evaluate whether any changes occur during the study period. Specific treatment aspects include: pain medications for OA (prescription and non-prescription), knee braces, walking aids, physical therapy sessions, and joint injections.

#### Participant feedback on IBET and standard PT arms

Following completion of the IBET and PT interventions, participants are asked a series of questions to assess which aspects were most and least helpful, usability of the IBET website, content of the PT sessions, and ways we may be able to improve the interventions.

### Data analyses

For the superiority hypotheses (H1, H3), analyses will be conducted on an intent-to-treat (ITT) basis. Patients will be analyzed in the arm to which they were randomized, regardless of adherence, using all available follow-up data [48]. Additional exploratory analyses focusing on alternative, more restrictive analytic cohorts (e.g., as treated) may be considered for the superiority hypotheses, to provide additional information about the impact of magnitude of exposure to the interventions. For non-inferiority hypotheses (H2, H4), the ITT analysis would not be the conservative approach. We will therefore perform analysis on both an ITT and as-treated basis [49, 50].

#### Descriptive statistics

Descriptive statistics, including graphical displays, will be used to summarize all study variables overall and by randomization arm. This will include individual and mean trajectory plots of the longitudinal outcome variables to understand their general trends over the study period. The variability and correlation structure of the longitudinal outcome variables will also be explored. All statistical analyses will be performed using the SAS (Cary, NC) software package.

#### Analysis of specific aim #1

##### H1 (Superiority)

Separate general linear mixed models (GLMMs) will be fitted to follow-up WOMAC scores as the dependent variables. An unstructured covariance matrix will be applied to take into account the within-patient correlation between the two follow-up repeated measures. The model will contain fixed effects for follow-up time (2 levels) and for intervention group (3 levels), as well as their interaction. The baseline score for each dependent variable will be included as a covariate, and the models will also be adjusted for enrollment site (stratification variable). The SAS MIXED procedure (Cary, NC) will be used to estimate the parameters in the model and test contrasts corresponding to each hypothesis at 4 months. Participants who are missing either follow-up measurement will still be included in the model under a ‘missing at random’ paradigm.

##### H2 (Non-Inferiority)

A non-inferiority margin of 5 points for mean WOMAC scores was chosen because it is reasonable and on the border of what would be considered a clinically important effect [51]. The null hypothesis in the non-inferiority framework is that IBET is inferior to standard PT in management of OA symptoms. This hypothesis will be tested through the adjusted contrast between the two interventions at 4 months from the mixed model specified above. Specifically, the 95 % CI of the estimated contrast will be examined, and if the upper limit of the interval is less than the threshold value of 5 points, non-inferiority of IBET to PT at 4 months will be concluded [50]. If non-inferiority is concluded, a test will also be conducted for superiority of IBET to PT at 4 months.

#### Analysis of specific aim #2

##### H3 (Superiority) and H4 (Non-Inferiority)

As described above, for each GLMM, the same testing/procedures as described above will be applied, but using the 12-month follow-up parameters rather than those for 4-months.

#### Analysis of specific aim #3

Patients may vary in their response to the intervention programs. This variation is known as heterogeneity of treatment effects (HTE). This aim will involve descriptive HTE analyses [52]. Patient age and baseline functional status are the 2 a priori defined characteristics for the initial focus of these analyses. Patient age will be managed as a continuous variable, and age categories (e.g., by decade) will also be explored. With respect to functional status, objective function test scores (described above) will be used, since this is a separate measure from our primary outcome (WOMAC). Individual and mean trajectory plots of the longitudinal outcome variables will be constructed by patient characteristics and intervention arm to understand their general trends over the study period. The general steps in this secondary analysis will be to add each of the patient characteristic main effects, as well as the corresponding interaction terms, to separate GLMMs, as defined above. The 3-way interactions (treatment*time*patient characteristic) will be tested to determine whether there is evidence of HTE for that characteristic. If statistical significance is found, then contrasts will be constructed to estimate the distinct intervention effects at each follow-up time for varying levels of the patient characteristic.

#### Missing data

Outcome values may be missing due to dropout, death, a missed interim assessment, or item non-response. The main analytic methodology for the primary outcomes, GLMMs via maximum likelihood estimation, implicitly accommodates missingness when it is due either to treatment, to prior outcome, or to other baseline covariates included in the model, defined as ‘missing at random’ [53]. Therefore, inferences will be valid even if there is differential dropout by intervention arm. If the missing values are determined not to be missing at random, then multiple imputation (MI) provides a framework for incorporating information about the missingness, while still preserving a parsimonious main treatment effect model [54], and is described as a significant advantage in recommendations from Panel on Handling Missing Data in Clinical Trials [55]. Depending on the type and scope of missing data, MI will be conducted via the SAS procedure PROC MI or the SAS macro IVEware (http://www.isr.umich.edu/src/smp/ive/). In this this situation, additional sensitivity analyses to explore missingness will be conducted, possibly including selection and pattern-mixture models [56].

#### Multiple comparisons

Although approaches to controlling statistically for multiple comparisons can vary widely, we have adopted a strategy whereby we aim to control the significance level at the two-sided .05 level (or, as applicable and correspondingly, the one-sided .025 level) separately for each hypothesis specified above. More specifically, the superiority hypothesis, H1, involves two formal comparisons (i.e., IBET vs. WL control, and, separately, standard PT vs. WL control); each will be conducted at the two-sided .025 significance level. The same approach will be utilized for the superiority hypothesis, H3. The non-inferiority hypothesis in H2 involves only one comparison (i.e., IBET vs. standard PT), so it will be tested at the full one-sided .025 significance level; this also applies separately to hypothesis H4. Adjustment to the significance level will not be performed for secondary outcomes.

### Sample size

The sample size estimate of *n* = 350 participants is based on H2, the non-inferiority hypothesis, as this is the most conservative [49, 57, 58], and on a randomization of three experimental groups in a 2:2:1 ratio [59]. Sample size calculations are based on this comparison and use methods appropriate for ANCOVA analyses, which are equivalent in terms of efficiency to our linear model in randomized trials [60]. For the non-inferiority test, the method is based on performing a one-sided two-sample *t*-test sample size calculation at the alpha = 0.025 level for the between-group difference at the 4 month time point, multiplying the variance by a factor 1-*ρ*^2^, where *ρ* represents the Pearson correlation coefficient between baseline and follow-up time point outcome measures [61]. This sample size is then adjusted to compensate for potential missing observations due to attrition. Based on data from our other OA studies, *ρ* was assumed as 0.6 for the WOMAC scores, along with a standard deviation (SD) of 17.5. An attrition rate of 10 % is assumed based on our prior research of behavioral interventions for OA of similar duration [62]. Under 80 % power, one-sided *α* = .025, SD = 17.5, *ρ* = 0.60, and a 10 % attrition rate by 4 months, 140 patients in each of the IBET and standard PT groups need to be enrolled at baseline to identify less than a 5 point difference in mean WOMAC scores between the two treatment groups. As originally proposed, for H1, with two-sided *α* = 0.05, SD = 17.5, *ρ* = .6, and approximately 10 % attrition rate by 4 months, there would be 80 % power to detect a moderate effect size of 0.35, corresponding to a 6.1 point difference (representing approximately 13 % improvement) in mean total WOMAC scores at 4 months for either of the two intervention groups separately compared to the WL control group. Using a two-sided *α* = .025 level to control for multiple comparisons within this hypothesis (as detailed above), the corresponding mean difference in WOMAC scores would increase to 6.75 points to maintain 80 % power. For the remaining secondary outcomes we will also be powered to detect a moderate 0.35 effect size difference for either of the two interventions separately compared to the WL control group at the two-sided *α* = 0.05 level.

## Discussion

As with all clinical trials, there are limitations to this study. Because of the nature of the IBET intervention, all study participants must have regular internet access. This limits generalizability of findings to patients with OA who do not have access. Internet and mobile device use is increasing rapidly, including among older adults. However, other types of home-based interventions should be considered for patients with OA who are not regular internet users. Another potential limitation is that study participants in each group may receive other OA treatments during the study period. This design was chosen for ethical considerations. Receipt of other OA treatments is being rigorously evaluated and will be considered in study analyses.

This project has several novel and important features. First, to our knowledge it is the first randomized clinical trial of an internet-based exercise program among patients with knee OA, across the spectrum of disease duration and severity. Pilot study results of this study were very promising [26], but a rigorous clinical trial is needed to further examine its effectiveness. Second, this study compares the IBET program not only to a wait list control group but also to standard PT, a treatment that has an established evidence base for knee OA [10–12]. The IBET program and standard PT differ in many aspects, but they share common goals of improving physical activity and function among patients with knee OA. By comparing the IBET program directly to PT, this study will help patients with OA and health care providers understand whether IBET can provide similar benefits to patients in terms of pain and functional outcomes. If benefits are similar, IBET could be an alternative management strategy to consider when patients with knee OA do not have access to or cannot afford PT. This type of internet-based exercise program could serve as a prototype for other musculoskeletal conditions. In addition, the study will evaluate whether some patient characteristics predict differential benefit from IBET or PT, providing guidance for referring patients to a treatment option that may be most appropriate for them. Third, this study takes a pragmatic approach, including participants who experience the spectrum of OA symptom severity and incorporating minimal exclusion criteria (e.g., those that may make exercise unsafe or confound study outcomes). This is important for generalizing findings to real-world clinical settings. A fourth important aspect of this study is the involvement of a diverse Stakeholder Panel, comprised of patients with knee OA, primary care and specialty physicians who treat patients with knee OA, physical therapists, and representatives of national organizations seeking to improve outcomes for people with OA. This group has made key contributions to the development of the study and its ongoing activities, with a view toward ensuring that study results will be of high relevance to patients with OA and their health care providers.

## Appendix

### Guidance for structure and content of physical therapy visits

1. Programs, both in the clinic and at home, should be comprehensive and functional, focusing on core and lower body function, but can be tailored to meet the functional abilities, needs and deficits of each participant.
2. Each visit should emphasize therapeutic exercise and include muscle strengthening, stretching/flexibility/range of motion, and aerobic exercise.
3. Education on activity pacing, joint protection and pain management
4. A home program should be recommended during the 1^st^ visit and should be progressed over the course of treatment.
5. Home programs should emphasize the following:
  - Strengthening Exercises
    i. Recommend performing strengthening exercises 2-3 times per week
    ii. Include functional exercises, such as gait or stair training and neuromuscular education
  - Stretching/flexibility/range of motion Exercises
    i. Recommend performing range of motion exercises daily
  - Aerobic Exercises
    i. Promote “lifestyle” physical activity
    ii. Encourage moderate intensity exercise
    iii. Episodes of activity should last at least 10 minutes, if the participant is able
    iv. Episodes should be spread out throughout the week with a long-term goal of working up to a total of 150 minutes of activity per week
    v. Aerobic exercise can be weight-bearing, reduced weight-bearing or non-weight-bearing.
6. Modalities for pain management can be included during the clinic visit and as part of the home program. Modalities should be used conservatively, taking no more than 25% of the time of each clinic visit.
7. If appropriate, manual therapy and/or patellar taping can be provided during the clinic visit.
8. Shoes should be assessed during the first visit, and shoe recommendations should be provided, if appropriate.
9. If limb length inequality or frontal plane knee malalignment is suspected, treatment with shoe lifts or shoe wedges, respectively, should be attempted.

## Abbreviations

OA: Osteoarthritis
PT: Physical Therapy
IBET: Internet-based Exercise Training
WL: Wait List
WOMAC: Western Ontario and McMasters Universities Osteoarthritis Index
mSF-WOMAC: Modified Short Form of the Western Ontario and McMasters Universities Osteoarthritis Index
PRIME-MD: Primary Care Evaluation of Mental Disorders
PASE: Physical Activity Scale for the Elderly
ITT: Intent-To-Treat
GLMM: General Linear Mixed Model
HTE: Heterogeneity of Treatment Effects
MI: Multiple Imputation
ANCOVA: Analysis of Covariance
UNC: University of North Carolina at Chapel Hill
